# Draft Genome Sequence of a Metabolically Diverse Antarctic Supraglacial Stream Organism, *Polaromonas* sp. Strain CG9_12, Determined Using Pacific Biosciences Single-Molecule Real-Time Sequencing Technology

**DOI:** 10.1128/genomeA.01242-14

**Published:** 2014-12-04

**Authors:** Heidi J. Smith, Christine M. Foreman, Thiruvarangan Ramaraj

**Affiliations:** a Department of Land Resources and Environmental Sciences, Montana State University, Bozeman, Montana, USA; b Department of Chemical and Biological Engineering, Montana State University, Bozeman, Montana, USA; c Center for Biofilm Engineering, Montana State University, Bozeman, Montana, USA; d National Center for Genome Resources (NCGR), Santa Fe, New Mexico, USA

## Abstract

*Polaromonas* species are found in a diversity of environments and are particularly common in icy ecosystems. *Polaromonas* sp. strain CG9_12 is an aerobic, Gram-negative, catalase-positive, white-pigmented bacterium of the *Proteobacteria* phylum. Here, we present the draft genome sequence of *Polaromonas* sp. strain CG9_12, isolated from an Antarctic supraglacial stream.

## GENOME ANNOUNCEMENT

Organisms from the genus *Polaromonas* are receiving increased attention due to their environmental ubiquity. The genus *Polaromonas* was originally proposed to describe marine, psychrophilic, Antarctic organisms (1), and *Polaromonas* sequences have been retrieved from a wide variety of icy environments (2–6). It is hypothesized that the widespread distribution of phylotypes might be due to the presence of genetic *hipA* machinery, which produces dormant cells (7). Organisms from the *Polaromonas* genus are also studied for their role in pollutant degradation. Currently, there are only five whole genomes publicly available, four isolated from the sediment/groundwater environment (8–10) and one from an Arctic glacier (6).

Recently, *Polaromonas* organisms were shown to be capable of utilizing a variety of energy sources from recalcitrant organic compounds (11) to arsenic (12), dichloroethane (10), and hydrogen (13). As a result of their metabolic diversity, they have been described as having an opportunistic metabolism (7). Recent studies suggest that polaromonads are widespread, but still there is relatively little known about their metabolic strategies and environmental roles. To gain further insight into dispersal and metabolic strategies, additional *Polaromonas* genomic sequences are necessary.

*Polaromonas* sp. strain CG9_12 was isolated from a supraglacial stream on the Cotton Glacier, Antarctica (77°07′S, 161°50′E). The organism was isolated on R2A agar medium incubated in the dark at 4° C for 12 days. *Polaromonas* sp. strain CG9_12 is a psychrotolerant, aerobic, rod-shaped, Gram-negative, catalase-positive, white-pigmented organism, which has *hipA* dormancy genes. Genomic DNA was isolated following standard cetryltrimethylammonium bromide (CTAB) isolation protocols (http://www.jgi.doe.gov).

Sequencing was performed on a Pacific Biosciences (PacBio, Menlo Park, CA) RSII instrument (14). A SMRTbell library was constructed with 5 µg input DNA using the PacBio low-input 10-kbp protocol. The library was then loaded onto two single-molecule real-time (SMRT) cells and sequenced using P4 polymerase and C2 chemistry with 180-minute movie times. Sequencing yielded a total of 281,150 reads with mean read length of 3.2 kbp, totaling 900,510,276 bp (~150× coverage). *De novo* assembly was carried out using the hierarchical genome assembly process (HGAP) protocol from SMRT Analysis v2.0, including consensus polishing with Quiver (15, 16). The final assembly consists of seven contigs with a total genome size of ~4.9 Mbp. Approximately 91% of the genome is contained within one large 4.5-Mbp contig. Remaining sequences were divided into six smaller contigs ranging from 19 to 183 kbp. A total of 4,975 candidate protein-coding genes were predicted with a total G+C content of 60.1%. The small-subunit rRNA gene sequences had 99% sequence identity to *Polaromonas glacialis* strain Cr4-12, which was isolated from an alpine glacier cryoconite (GenBank accession number NR109013). SMRT DNA modification detection analysis (17) detected three 6-methyladenine modified motifs, 5′-G**A**CN_7_AATC-3′, 5′-G**A**TTN_7_GTC-3′, and 5′-TGAGT-3′, exhibiting >99% confidence of their being methylated in the genome. Another motif, 5′-GACATG-3′, detected in the genome with a high (>94%) confidence level, was categorized as an unknown modification.

### Nucleotide sequence accession numbers.

The draft whole-genome sequences have been deposited at DDBJ/EMBL/GenBank under BioProject number PRJEB6335 and the whole-genome accession numbers CCJP01000001 through CCJP01000007. The version described in this paper is the first version.
